# Restitution and genetic differentiation of salmon populations in the southern Baltic genotyped with the Atlantic salmon 7K SNP array

**DOI:** 10.1186/s12711-015-0121-9

**Published:** 2015-05-06

**Authors:** Anita Poćwierz-Kotus, Rafał Bernaś, Matthew P Kent, Sigbjørn Lien, Egidijus Leliűna, Piotr Dębowski, Roman Wenne

**Affiliations:** Institute of Oceanology, Polish Academy of Sciences, Sopot, 81-712 Poland; Department of Migratory Fishes in Gdansk 80-298, Inland Fisheries Institute, Olsztyn, 10-719 Poland; Centre for Integrative Genetics (CIGENE), Norwegian University of Life Sciences, Ås, 1432 Norway; Institute of Ecology of Nature Research Centre, Vilnius, 08412 Lithuania

## Abstract

**Background:**

Native populations of Atlantic salmon in Poland, from the southern Baltic region, became extinct in the 1980s. Attempts to restitute salmon populations in Poland have been based on a Latvian salmon population from the Daugava river. Releases of hatchery reared smolts started in 1986, but to date, only one population with confirmed natural reproduction has been observed in the Slupia river. Our aim was to investigate the genetic differentiation of salmon populations in the southern Baltic using a 7K SNP (single nucleotide polymorphism) array in order to assess the impact of salmon restitution in Poland.

**Methods:**

One hundred and forty salmon samples were collected from: the Polish Slupia river including wild salmon and individuals from two hatcheries, the Swedish Morrum river and the Lithuanian Neman river. All samples were genotyped using an Atlantic salmon 7K SNP array. A set of 3218 diagnostic SNPs was used for genetic analyses.

**Results:**

Genetic structure analyses indicated that the individuals from the investigated populations were clustered into three groups i.e. one clade that included individuals from both hatcheries and the wild population from the Polish Slupia river, which was clearly separated from the other clades. An assignment test showed that there were no stray fish from the Morrum or Neman rivers in the sample analyzed from the Slupia river. Global F_ST_ over polymorphic loci was high (0.177). A strong genetic differentiation was observed between the Lithuanian and Swedish populations (F_ST_ = 0.28).

**Conclusions:**

Wild juvenile salmon specimens that were sampled from the Slupia river were the progeny of fish released from hatcheries and, most likely, were not progeny of stray fish from Sweden or Lithuania. Strong genetic differences were observed between the salmon populations from the three studied locations. Our recommendation is that future stocking activities that aim at restituting salmon populations in Poland include stocking material from the Lithuanian Neman river because of its closer geographic proximity.

**Electronic supplementary material:**

The online version of this article (doi:10.1186/s12711-015-0121-9) contains supplementary material, which is available to authorized users.

## Background

Atlantic salmon (*Salmo salar,* L) has considerable economic, social and environmental importance since it contributes to global and local economies through aquaculture, wild stock fisheries and recreational sport [1]. However, anthropogenic pressure and environmental factors have reduced natural populations of salmon, and thus, fisheries management has developed strategies for fish stocking in Pacific and Atlantic regions [2-5].

A range of potential ecological and genetic problems are associated with the release of artificially produced fish into wild populations [6,7]. Genetic and phenotypic differences may exist between hatchery fish and wild fish, which may affect how stocked and wild fish interact. Hatchery fish experience altered selection pressures i.e. high juvenile density and abundance of food may select for behavioral and physiological traits that are disadvantageous in natural conditions [6]. Thus, multi-generation hatchery stocks are likely to differ more from wild fish than first-generation stocks for which most of the changes are probably due to environmental effects. The use of non-native fish for stocking can cause the introduction of novel genetically-based features into the wild population and can break up co-adapted gene complexes that may lead to out-breeding depression [8].

The Baltic salmon is geographically and genetically distinct from other lineages of Atlantic salmon [9,10]. Most of the original genetic diversity of the wild Baltic salmon has been lost. Only 25 of the 90 original stocks have survived [11]. Juvenile salmon migrate out to the Baltic Sea to feed and grow and then migrate back to rivers to reproduce. Natal-river homing facilitates local adaptation because salmon return to environments with favorable spawning conditions [12]. The main reasons for the decline of wild stocks are hydroelectric constructions and over-exploitation of fish stocks in the Baltic Sea area [13].

To compensate for the decline of salmon populations, approximately 50 million salmon juveniles originating from hatcheries have been released by the Baltic countries, mainly Sweden and Finland, over the last ten years [14]. A survey of the genetic differentiation of contemporary Baltic salmon populations, as part of the North Atlantic range, has been undertaken using a range of genetic markers [10,15]. However, salmon from the southern Baltic are under-represented in these studies [16].

In Poland, the native Atlantic salmon has disappeared from all rivers i.e. first from the upper Vistula river in the 1950s, then from Pomeranian rivers in the 1960s, and finally the Drava river (Odra basin) by the end of the 1980s [17]. Year 1968 was most crucial since the Włocławek Dam power plant started operating on the Vistula river. Because of the complete extinction of salmon in Poland, a restitution program was initiated based on the Latvian population from the Daugava river [18]; it was not possible to use stocking material from the geographically closer Lithuanian Neman river at that time because of its small population size. Genetic studies based on allozymes [19] and microsatellites [16,20] revealed that the Latvian salmon population belonged to the Eastern group of Baltic salmon stocks. The first stocking of Polish rivers began in 1986 when 840 salmon smolts (1+) were released. The restitution program has continued to this day and can be considered as only moderately successful. Effective natural spawning has been evidenced by the presence of a limited number of wild parr found only in the Slupia river [21,22]. In addition to released fish, fish straying from other rivers or their descendants may have contributed to the restituted population. Our aim was to investigate the genetic differentiation of salmon populations in the southern Baltic using a 7K SNP (single nucleotide polymorphism) array in order to assess the impact of salmon restitution in Poland. Salmon that were introduced and naturalized in the Slupia river were compared with their source stocks at Polish hatcheries and subsequently with two neighboring populations from Sweden and Lithuania.

## Methods

### Sampling, DNA isolation and SNP (single nucleotide polymorphism) genotyping

**Figure 1:**
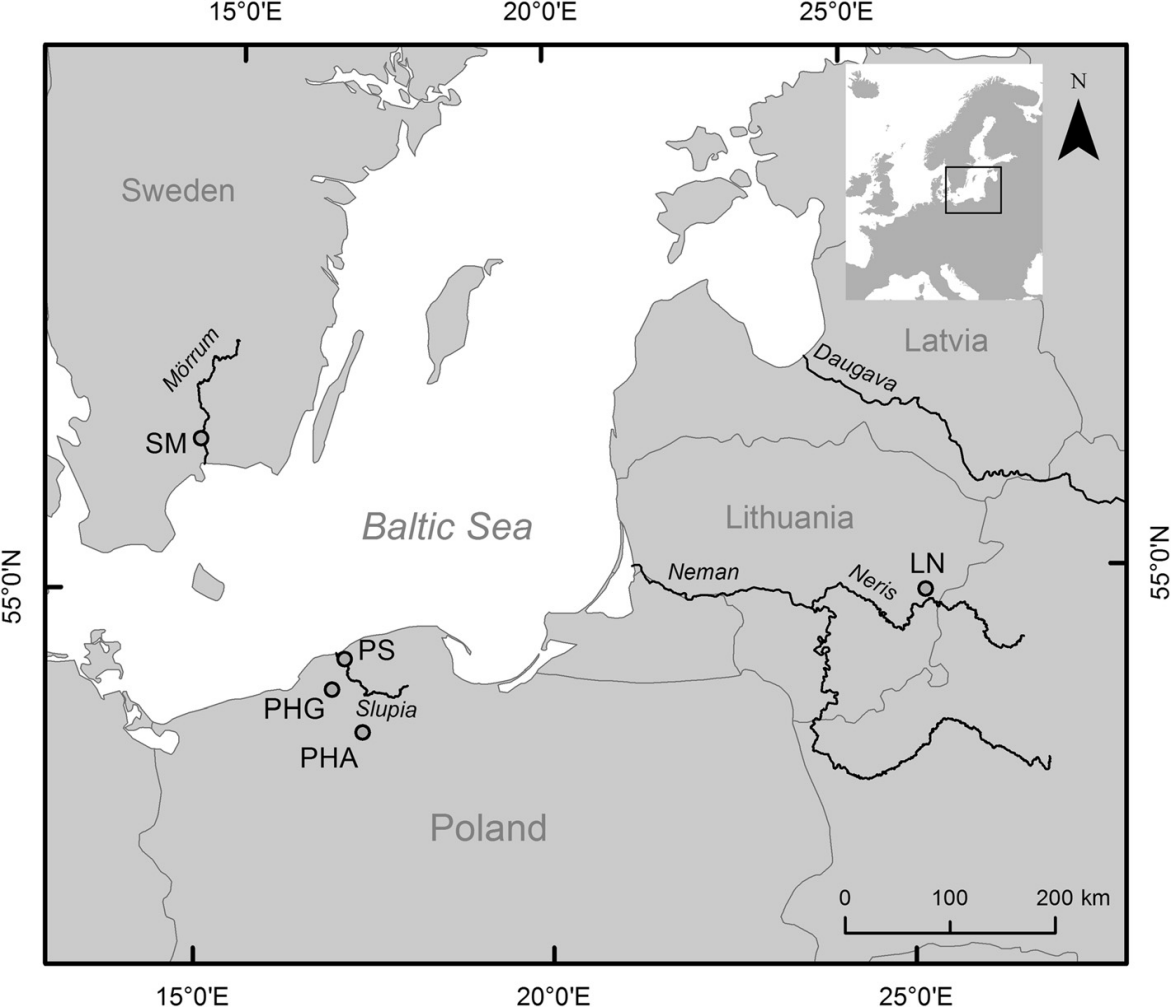
Map of salmon sample sites in the Southern Baltic. PS = Poland, Slupia river; PHA = Poland, hatchery Aquamar; PHG = Poland, hatchery Gabriel; SM = Sweden, Morrum river; LN = Lithuania Neman river.

Salmon samples from 28 individuals at each location were collected in 2011 from five locations in the southern Baltic: in Poland, wild parr from the Slupia river (PS) and parr from the two hatcheries Gabriel-Żelkówko (PHG) and Aquamar-Miastko (PHA); in Sweden, wild smolts from the Morrum river (SM); and in Lithuania, wild adults from the Neris (LN) river that is a tributary of the Neman river (Figure 1). Wild juvenile salmon from natural reproduction were electrofished in the Slupia river. DNA was extracted from fin clip samples with the Qiagen DNeasy 96 Blood & Tissue kit according to the manufacturer’s recommendations. DNA quality was analyzed on 1.0% agarose gels. DNA was quantified with a NanoDrop device and diluted to final concentrations of 50 to 100 ng/μL. DNA genotyping was performed using the Atlantic salmon Illumina 7K SNP chip [10] at the Centre for Integrative Genetics (CIGENE) in Norway.

### Validation of SNPs

Of the 5568 genotyped SNPs, 1640 were rejected as they failed in one or more of the following criteria: multi-site SNPs, paralogous SNPs, monomorphic SNPs and SNPs with null alleles. Mitochondrial SNPs were also excluded from the analysis. An accepted threshold of missing data rate was established at 80%, which excluded 13 SNPs. In total, 488 SNPs were found monomorphic for all the analysed populations and were excluded from further analysis. Analysis of SNP allele frequency revealed that 209 SNPs had a MAF (minor allele frequency) less than 0.01 and they were removed. Finally, 3218 polymorphic SNPs remained for the analyses.

### Analysis of SNP polymorphism, genetic structure and phylogenetic relationships

The number of polymorphic SNPs and their observed and expected heterozygosity (*Ho* and *He*) were calculated using the Arlequin 3.5.1.2 software [23] with the Markov chain exact probability test with a chain length of 1 000 000 and 100 000 dememorization steps. To adjust the P value for each pair in multiple testing, Bonferroni corrections were applied. For each population, allele frequencies were calculated using the Arlequin 3.5.1.2 software and MAF were estimated using Excel spreadsheet formulas. We also used the Arlequin software to perform an analysis of molecular variance (AMOVA) with 10 000 permutations to estimate variance components between Atlantic salmon populations and between individuals within populations by applying the bootstrapped F_ST_ estimator of Weir and Cockerham [24]. To estimate within-population diversity, the average number of pairwise differences was calculated using Arlequin. Pairwise F_ST_ was estimated in two datasets both for all SNPs and only outlier SNPs. The first dataset comprised the full geographic distribution i.e. the Latvian-Polish PL pooled population (i.e. pooled PS, PHA and PHG populations), the Lithuanian LN and Swedish SM populations, and the second dataset contained only the Polish PS, PHA and PHG populations.

Clustering of salmon populations was examined using Structure 2.0 software [25] assuming K equal to 1 to 8. Choosing a K value greater than 5 (the number of populations) was justified by the probability of identifying sub-populations among the studied populations. The Structure algorithm included the admixture model and correlated allele frequencies. Five iterations of each K value were conducted with 200 000 Markov chain Monte-Carlo (MCMC) iterations and 100 000 burn-in iterations. CLUMPP v. 1.1.1 [26] was applied to avoid the potential effect of generating several distinct solutions for the estimated cluster membership coefficients in spite of identical initial conditions The average cluster membership was calculated using the LargeK Greedy algorithm.

Distruct v. 1.1 [27] was used to visualize the results from the CLUMPP analysis by generating bar plots that depict the clustering results with the highest probability under the model. Plots show population and individual levels of stratification.

Structure Harvester [28] was used to determine the appropriate K value following Evanno et al. [29]. Principal Coordinates Analysis (PCoA) was performed in GenAlex [30,31] to visualize the relationships between populations. Phylogenetic relationships between the salmon populations were constructed using the POPULATIONS software version.1.2.32 [32] with the Neighbour-Joining (NJ) method and Nei’s standard genetic distance Ds [33]. Bootstrapping was carried out with 5000 replicates over loci, using the grouped population option.

To determine the most likely origin of all 140 salmon individuals, assignment tests were conducted using GeneClass2 [34] with the allele frequency-based method [35]. This allowed us to identify potential migrants or their descendants.

### Detection of outlier SNPs

The hierarchical island model with 100 000 simulations implemented in Arlequin was used to detect outlier SNPs. SNPs that had F_ST_ values for a given value of heterozygozity higher than expected on the basis of neutral variation were considered. SNPs that had F_ST_ values that were outside the 99% quantile based on coalescent simulations were considered as candidates for diversifying selection [36]. Significance of F_ST_ distributions for regular and outlier SNPs was tested using the Bayesian estimation software (BEST) [37] that supersedes the t test [37].

## Results

### Analysis of the genetic polymorphism and diversity of Salmo salar populations

The five salmon samples used in this work represent wild and hatchery populations. One hundred and forty individuals were genotyped using 5568 SNPs, of which 3218 were diagnostic markers. The number of polymorphic SNPs for each population varied and ranged from 2461 for the LN population to 3030 for the SM population. For the PS wild population and the PHA and PHG populations, the numbers of polymorphic SNPs were similar i.e. 2645, 2654 and 2620, respectively (Table 1). Mean numbers of SNP alleles were also congruent for the PS, PHA and PHG populations and the lowest and highest values were observed for LN (1.765) and SM (1.942) populations, respectively.

**Table 1:** **Levels of genetic diversity for five salmon populations from the southern Baltic Sea**

**Population**	**Nb individuals**	**Nb polymorphic loci**	**Mean nb alleles**	***Ho***	***He***	Nb loci deviating from HWE*	**After Bonferroni correction**	**F**_**is**_
PHA	28	2654	1.825	0.330	0.327	23	5	−0.00517
PHG	28	2620	1.814	0.339	0.331	68	2	−0.02221
LN	28	2461	1.765	0.339	0.323	83	6	−0.04648
SM	28	3030	1.942	0.325	0.325	85	2	−0.00038
PS	28	2645	1.822	0.337	0.327	88	6	−0.02486

PHA = Polish Hatchery Aquamar-Miastko population; PHG = Polish Hatchery Gabriel-Żelkówko population; LN = Lithuanian Neman river population; SM = Swedish Morrum river population; PS = Polish Slupia river population; *P < 0.05. Bonferroni correction was applied.

Observed heterozygosities were similar for all populations and ranged from 0.325 for SM to 0.339 for PHG and LN populations and expected heterozygosities ranged from 0.323 for LN to 0.331 for PHG populations. In all cases, differences between *Ho* and *He* were relatively small, with the largest difference observed for the LN (0.016) population while for SM, it was null. Before Bonferroni correction, deviations from Hardy-Weinberg expectations ranged from 23 for the PHG to 88 for the PS populations (Table 1). After Bonferroni correction, only a few SNPs remained significant from two for PHG and SM to six for LN and PS populations. Overall, F_IS_ reached a value of −0.022 and was non-significant (P < 0.05).The average population specific F_IS_ estimated for each population was also non-significant (P < 0.05) (Table 1).

AMOVA was conducted for three scenarios: “Countries”, “Wild populations”, and “Polish populations” (Table 2). The largest amount of variance was found among individuals within each population. For the “Countries” scenario, overall genetic differentiation (F_ST_) was equal to 0.226 which indicates a high level of differentiation. For comparison, a test was performed for a scenario that comprised only the wild populations and overall F_ST_ was even higher and reached 0.242. Overall F_ST_ was lowest (0.016) for the scenario that comprised only the Polish populations.

**Table 2:** **Analysis of molecular variance (AMOVA) applying the F**_**ST**_ **estimator of Weir and Cockerham** [24] **calculated for three models**

	**Between populations**		**Between individuals within populations**	
	**Variance component**	**% variation**	**Variance component**	**% variation**
Countries	129.15	22.65	441.14	77.35
Wild populations	141.05	24.25	440.65	75.75
Polish populations	7.46	1.69	433.68	98.31

Countries = PL (pooled Polish-Latvian populations), LN (Lithuanian Neman river) and SM (Swedish Morrum river) populations; Wild populations = PS (Polish Slupia river), LN and SM populations; Polish populations = PHA (Polish Hatchery Aquamar-Miastko), PHG (Polish Hatchery Gabriel-Żelkówko) and PS populations.

All pairwise comparisons of genetic differentiation between populations were significant (P < 0.05) (Tables 3 and 4). Pairwise F_ST_ analysis of the various geographic locations showed that PL vs. SM (0.21) and SM vs. LN (0.27) were genetically differentiated, while PL vs. LN and PL vs. SM were not. When only the Polish populations were analyzed, PS vs. PHG and PS vs. PHA had nearly equal F_ST_ values at 0.02. The SM population was the most genetically differentiated while the three polish PS, PHG and PHA populations had very similar, but lower, levels of within-population differentiation than SM. Finally, the population that was the least genetically differentiated was LN.

**Table 3:** **F**_**ST**_ **values for pairwise comparisons of salmon PL, LN and SM populations based on geographical location (below the diagonal) and average number of pairwise differences within populations (on the diagonal)**

	**PL**	**LN**	**SM**
PL	*876.431*		
LN	0.218	*795.614*	
SM	0.217	0.275	*983.046*

PL = pooled Polish-Latvian; LN = Lithuanian Neman river; SM = Swedish Morrum river; all values were significant for a P value of 0.05.

**Table 4:** **F**_**ST**_ **values for pairwise comparisons of salmon PHA, PHG and PS Polish populations (below the diagonal) and average number of pairwise differences within populations (on the diagonal)**

	**PHA**	**PHG**	**PS**
PHA	*865.342*		
PHG	0.008	*865.480*	
PS	0.019	0.022	*864.433*

PHA = Polish Hatchery Aquamar-Miastko; PHG = Polish Hatchery Gabriel-Żelkówko; PS = Polish Slupia river; all values were significant for a P value of 0.05.

### Analysis of outliers

**Figure 2:**
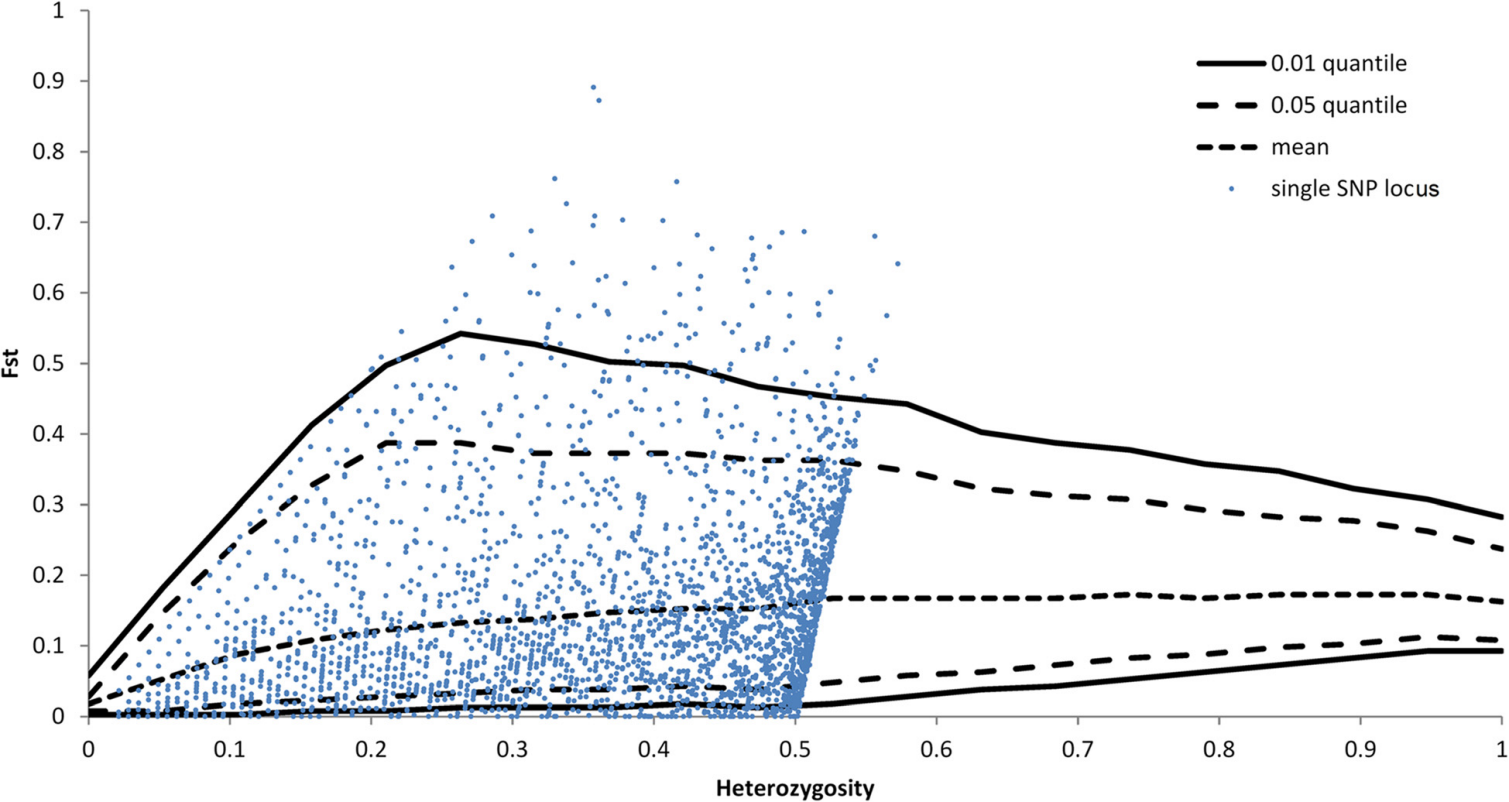
Analysis of outlier SNPs using a hierarchical model. SNPs that are above the 99% quantile of the simulation model (solid line) were considered as SNPs under potential selection. SNPs above the upper solid line were considered as candidates for divergent selection and those below the lower solid line as candidates for balancing selection. SNPs that are between the solid lines are neutral.

One hundred and twenty six SNPs for which F_ST_ values were outside the 99% quantile were identified as potential candidates for divergent selection (Figure 2). The global F_ST_ calculated for this set of 126 outlier loci for the pooled Polish populations was much higher i.e. 0.656 compared to that calculated for the 3218 polymorphic SNPs (0.226). Pairwise F_ST_ values increased significantly for all comparisons between populations in both analyses except for PS vs. PHA and PS vs. PHG for which F_ST_ values decreased non significantly (P = 0.073) from 0.019 to 0.014 for PS vs. PHA and from 0.022 to 0.015 for PS vs. PHG (Tables 5 and 6).

**Table 5:** **Pairwise estimates of F**_**ST**_ **calculated using the 126 outlier SNPs for salmon PL, LN and SM populations based on geographical location**

	**PL**	**LN**	**SM**
PL	-		
LN	0.679	-	
SM	0.680	0.589	-

PL = pooled Polish-Latvian; LN = Lithuanian Neman river; SM = Swedish Morrum river; all values were significant for a P value of 0.05.

**Table 6:** **Pairwise estimates of F**_**ST**_ **calculated using the 126 outlier SNPs for salmon PHA, PHG and PS Polish populations**

	**PHA**	**PHG**	**PS**
PHA	-		
PHG	0.021	-	
PS	0.014	0.015	-

PHA = Polish Hatchery Aquamar-Miastko; PHG = Polish Hatchery Gabriel-Żelkówko; PS = Polish Slupia river; all values were significant for a P value of 0.05.

### Analysis of population genetic structure and genetic relationships between populations

**Figure 3:**
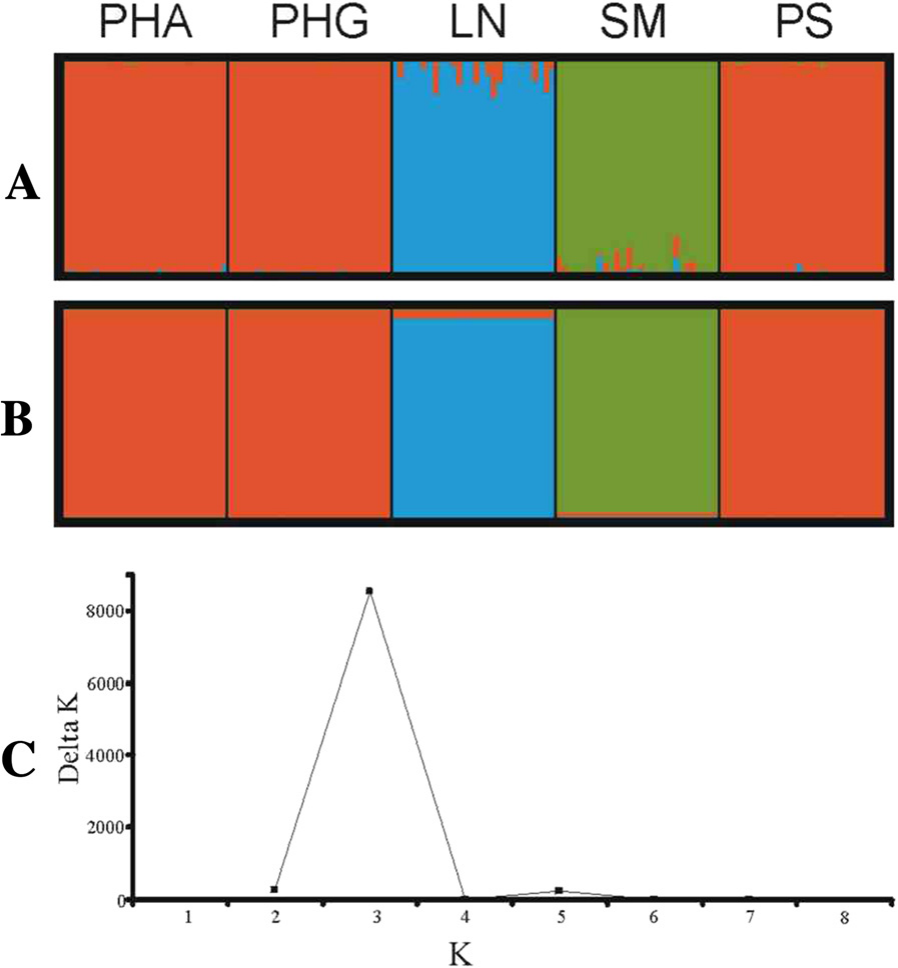
Population structure of five salmon populations using model-based Structure software and a dataset of 140 individuals and 3218 SNPs. The results were averaged by CLUMPP software and plots were generated by Distruct. **A** = individual level; **B** = population level; **C** = plot obtained from Structure Harvester for determining K.

Bayesian clustering methods were applied to examine genetic relationships between the five salmon populations and provided information about the assignment of particular individuals to groups based on their genetic similarity. The results obtained using the Evanno method [29] indicated that the mean log likelihood against K showed a plateau at K = 3 and the maximum value of ΔK was for K = 3 (ΔK = 8521) (Figure 3C). At K = 3, the three Polish PS, PHA and PHG populations were separated from the Lithuanian LN and Swedish SM populations (Figure 3).

Results from the assignment test showed that 85.00% of all individuals were assigned to the population they were sampled from (Table 7). The frequency of self-assignment varied from 60.17% for PHG to 100% for LN and SM. The percentage of correctly assigned individuals from the PS population was 78.61% while 20.43% and 0.95% of the PS individuals were assigned to the breeding PHA and PHG populations, respectively. However, individuals from these hatchery stocks were more mixed with each other than with PS itself. The main reason for such high similarity is that salmon eggs from PHA have been transferred to PHG at certain times over the last decade. 83.04% of the individuals from the PHA population were self-assigned while 16.98% were assigned to PHG, and 60.17% of the individuals from the PHG population were self-assigned while 39.82% were assigned to PHA. Assignment tests indicated that among all the individuals investigated from the Polish populations, no genotypes from the Swedish (Morrum) or Lithuanian (Neman) samples were observed.

**Table 7:** **Results of the assignment tests computed using GeneClass2 software based on a frequency method** [34]

	**PHA**	**PHG**	**LN**	**SM**	**PS**
**PHA**	83.04%	16.98%	0%	0%	0%
**PHG**	39.82%	60.17%	0%	0%	0%
**LN**	0%	0%	100%	0%	0%
**SM**	0%	0%	0%	100%	0%
**PS**	20.43%	0.95%	0%	0%	78.61%

Results are presented using the percent score of the most likely source population (threshold P < 0.05); PHA = Polish Hatchery Aquamar-Miastko; PHG = Polish Hatchery Gabriel-Żelkówko; LN = Lithuanian Neman river; SM = Swedish Morrum river; PS = Polish Slupia river.

Individual assignment results were consistent with the results of the pairwise F_ST_ analysis: LN and SM populations showed the highest pairwise F_ST_ values and had a self-assignment rate of 100%. Similar relationships were observed with both PCoA and Structure analysis [See Additional file 1: Figure S1].

**Figure 4:**
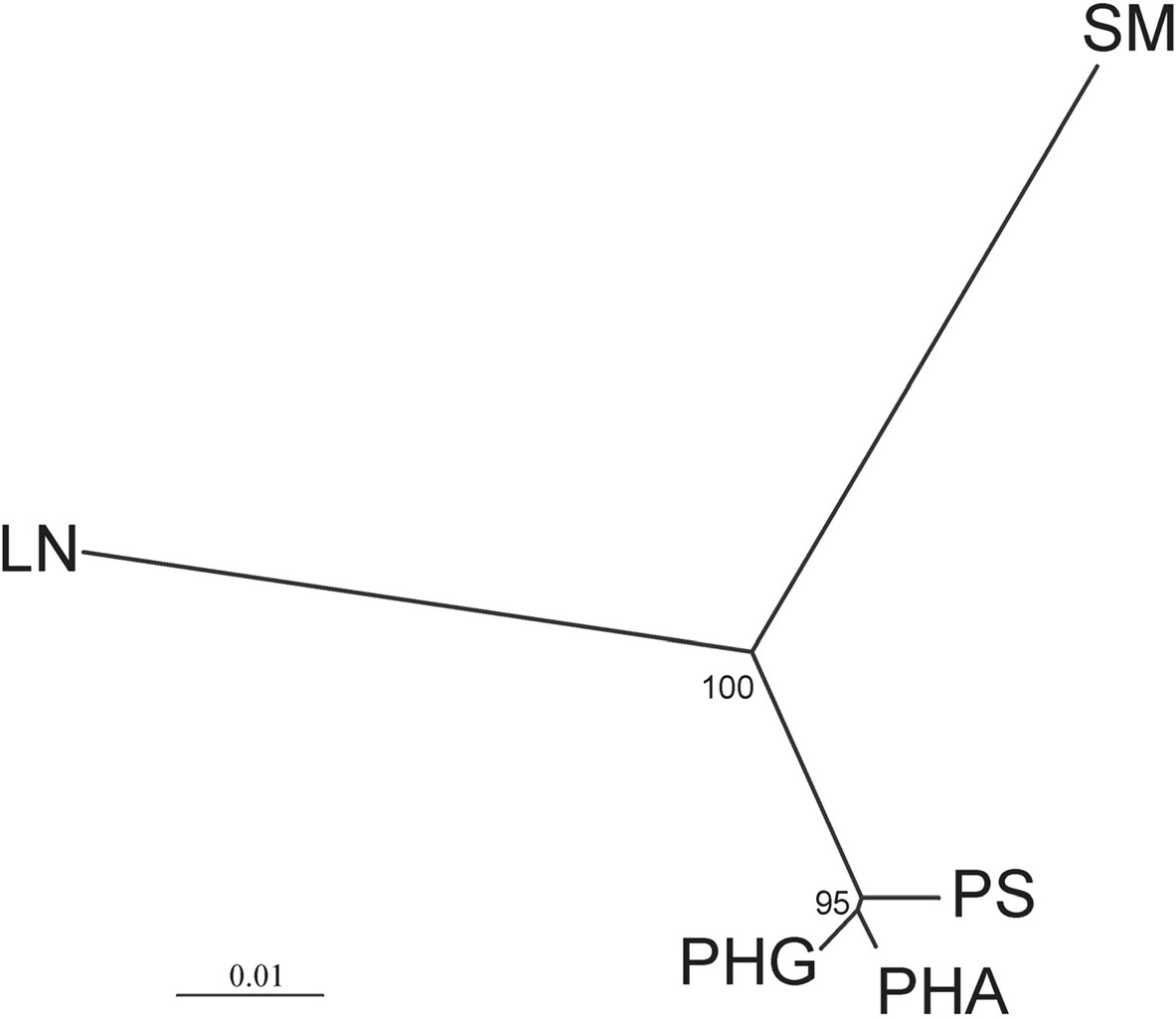
A neighbor-joining tree constructed using Nei’s distances among the five salmon populations and generated by POPULATIONS v.1.2.32 [32] software. Bootstrap probabilities are shown on the tree.

Genetic relationships between salmon populations based on Structure analysis were consistent with the results obtained from the phylogenetic analysis. A neighbour-joining (NJ) tree was constructed and the branches were supported by high bootstrap values (Figure 4). The NJ method showed that the genotypes investigated belonged to three major clusters: cluster (1) included all genotypes from the Lithuanian LN population, cluster (2) all genotypes from the Swedish SM population and cluster (3) all genotypes from the three Polish populations PS, PHA and PHG.

## Discussion

In this study, a 7K SNP microarray was used to analyze the genetic relationships between hatchery and wild salmon populations from the southern Baltic. The Polish wild population from the Slupia river (PS) and the two hatchery Polish populations PHA and PHG formed one clade. However, the PS individuals that were caught were born in the Slupia river where is located a recovering and naturally reproducing salmon population. Bayesian analysis and assignment tests showed that wild specimens sampled from the Slupia river were not the progeny of fish straying from Sweden or Lithuania but were the progeny of fish released from the hatcheries that were established by using imported stocking material from the Daugava river. Salmon eggs have been imported several times i.e. in 1985, 1987 and each year between 1994 and 1999 [17]. Previously, Popović [18] had already reported the similarity between Polish hatchery populations, including PHA and PHG, and the population from the Daugava river.

F_IS_ estimates for all Polish populations revealed that the genetic diversity of the Polish salmon does not seem to be affected by inbreeding. Therefore, the Polish hatchery Aquamar (PHA) stock consisting of a large number of specimens (about 700 females and 300 males) may be sufficient to preserve a hatchery strain against unfavorable factors such as inbreeding effects, genetic drift and loss of genetic diversity [18,37,38]. No inbreeding was observed for the other populations either. However, because sample size was small (28 specimens per hatchery) and SNPs were mainly biallelic, F_IS_ values need to be interpreted with caution since negative selection processes or domestication occurring in hatchery stocks cannot be excluded. In spite of the observed small, but significant, differences between the wild PS population and PHA and PHG hatchery stocks, the wild population was closely related to the stocking material.

The three Polish populations, PS, PHA and PHG separated well from the Lithuanian LN and Swedish SM populations. The results obtained from the genetic structure analysis suggested that the most significant subdivision is the geographic subdivision. The Polish populations irrespective of the site of sampling had the same ancestral population, which was confirmed by the results of NJ reconstruction and PCoA.

The stocking material used in the two sampled hatcheries in Poland came from Latvia (Daugava river) as eyed eggs and were imported each year between 1994 and 1999. Initially, the stocking material was reared and released at the smolts stage and later, an own-hatchery stock, located in Aquamar, was created. Currently, all stocking material in the Slupia basin is based on releases of smolts that are marked by clipping the small adipose fin near the tail. According to the results of studies on scales and tagging experiments, the rate of potential straying of Baltic salmon is relatively low (on average 4%) and the risk of contamination for neighboring native populations is near zero [39].

Based on microsatellite analyses [16], salmon from the Daugava river in Latvia represent eastern Baltic populations while the salmon sampled from the Morrum river in Sweden represent the southern Baltic group. Our results show that the population from the Neman river in Lithuania constitutes a third clade that is clearly separated from the other populations. This native population could be closely related to the extinct Polish salmon populations. It is recommended that future stocking activities that aim at restituting salmon populations in Poland, include material from the Neman river because of its closer geographic proximity.

However, in this study, the lowest genetic variability was observed for the LN population, which may be the result of a large reduction in effective population size in the past. Therefore, the genetic quality of this new potential stocking material from the Neman river needs to be analyzed to assess the genetic consequences on the recently established salmon population in the Słupia river in Poland prior to any releases.

The LN population is characterized by the smallest number of polymorphic SNPs and smallest mean number of alleles. In contrast, among all analyzed populations, diversity measures were greatest for the SM population. The current annual wild production of smolts is about 50 000 per year in the Neman and 60 000 in the Morrum rivers [39]. Both salmon populations from Morrum and Neman rivers have been classified as “wild” according to HELCOM (Baltic Marine Environment Protection Commission - Helsinki Commission) indicators. However, the Neman salmon is considered as more threatened because the size of its reproductive population is smaller. The Polish Slupia population has been described as “mixed” with studies reporting some annual wild rearing and continuous releases of reared fish [22,39]. Genetic differentiation between these populations could potentially have biological relevance by reflecting local adaptation or diversification of quantitative traits. Management based on the specificities of each river has been recommended by HELCOM [22] and by participants of The Baltic Salmon Symposium and Workshop held at the Stockholm University in February 9–10, 2012.

Previous research showed that Salmonidae, including sea trout (*Salmo trutta* m. *trutta*) and Atlantic salmon, underwent a bottleneck event [40,41]. The low level of diversity can be explained by this early bottleneck in the salmon populations of Lithuanian rivers. These populations originate from different evolutionary lineages related to the existence of distinct refugia. It has been suggested that the Baltic Sea was colonized from up to three distinct refugia: the Gulf of Bothnia from an Atlantic refugium, the Gulf of Finland from an eastern ice lake refugium and the southern Main Basin from a refugium that was presumably located in the basin of Neman, Vistula, Odra and Elbe rivers [16,42].

## Conclusions

In this study, we exploited a high-throughput SNP microarray technology that provided extensive information on the polymorphism of nuclear DNA in populations of salmon in the Southern Baltic. Salmon stocking material imported to Poland from Latvia (Daugava river) differed from southern Sweden and Lithuanian populations. Wild smolts from the restituted population in the Slupia river originated from fish released from hatchery stocks. No stray fish or progeny of fish straying from Sweden or Lithuania were found in the restituted population of the Slupia river. Our recommendation is that future stocking activities that aim at restituting salmon populations in Poland, include stocking material from the Neman river because of its closer geographic proximity.

## Additional file

Additional file 1: Figure S1. Principal coordinates analysis (PCoA) based on all SNPs. Principal coordinates analysis (PCoA) showed that axes 1 and 2 explain 41.60% and 36.45% of the total genetic variation, respectively. The analysed salmon individuals formed three clusters: the first (LN population) and second clusters (SM population) were clearly separated from the third cluster that consisted of individuals from the three Polish populations. Black triangle = Lithuanian Neman river (LN); empty circle = Swedish Morrum river (SM); gray diamond = Polish hatchery Aquamar (PHA); empty square = Polish hatchery Gabriel (PHG); grey star = Polish Slupia river (PS).
