# Supplementary figures and images for: Restitution and genetic differentiation of salmon populations in the southern Baltic genotyped with the Atlantic salmon 7K SNP array
Source: Genet Sel Evol. 2015 May 6;47(1):39. doi: 10.1186/s12711-015-0121-9 (PMC4421911; doi:10.1186/s12711-015-0121-9)

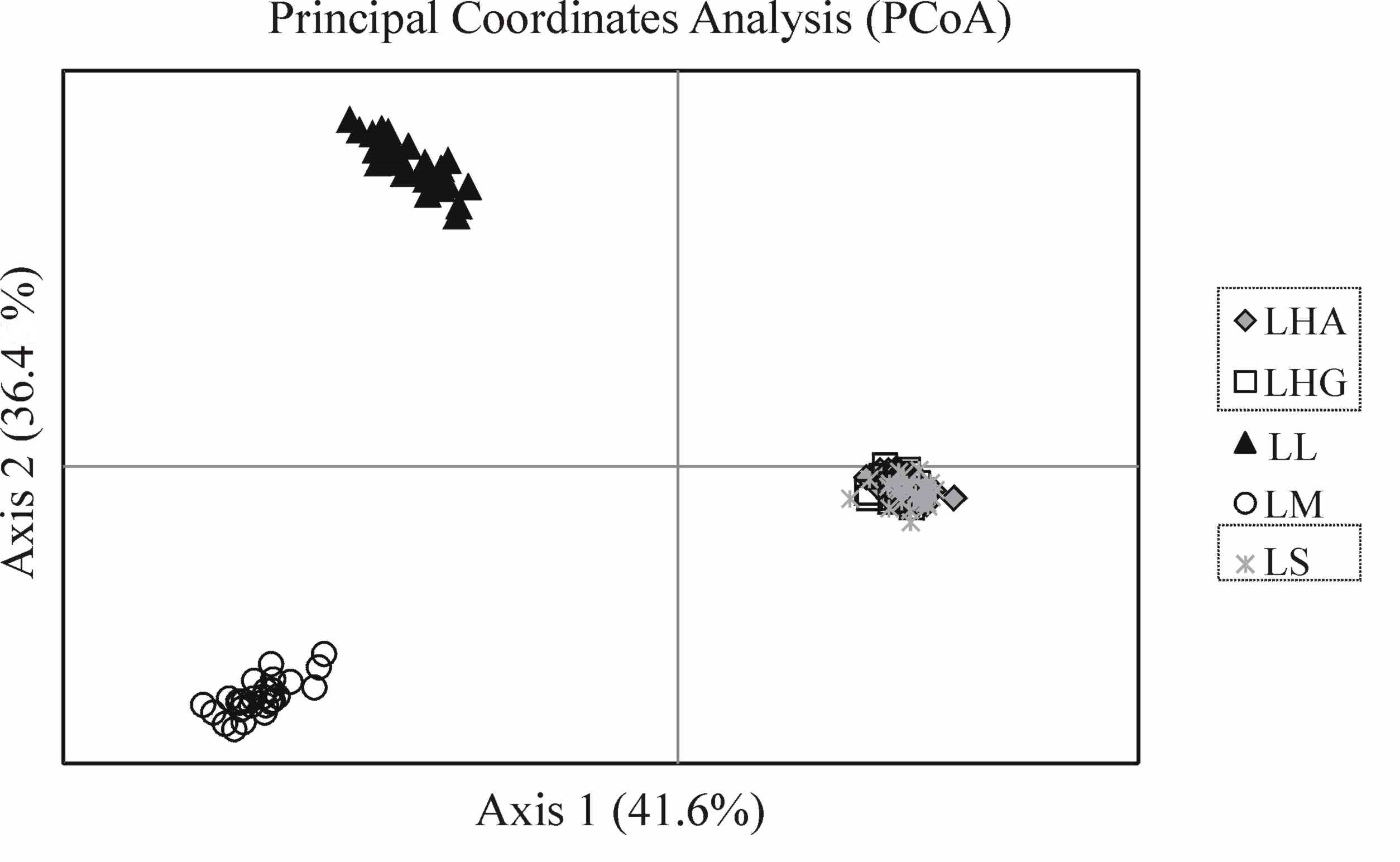

Supplement: Additional file 1: Figure S1. — Principal coordinates analysis (PCoA) based on all SNPs. Principal coordinates analysis (PCoA) showed that axes 1 and 2 explain 41.60% and 36.45% of the total genetic variation, respectively. The analysed salmon individuals formed three clusters: the first (LN population) and second clusters (SM population) were clearly separated from the third cluster that consisted of individuals from the three Polish populations. Black triangle = Lithuanian Neman river (LN); empty circle = Swedish Morrum river (SM); gray diamond = Polish hatchery Aquamar (PHA); empty square = Polish hatchery Gabriel (PHG); grey star = Polish Slupia river (PS). [file 12711_2015_121_MOESM1_ESM.jpeg]
